# Complete Genome Sequence of Pseudorabies Virus Reference Strain NIA3 Using Single-Molecule Real-Time Sequencing

**DOI:** 10.1128/genomeA.00440-16

**Published:** 2016-05-26

**Authors:** Elisabeth Mathijs, Frank Vandenbussche, Sara Verpoest, Nick De Regge, Steven Van Borm

**Affiliations:** a Molecular Platform, Veterinary and Agrochemical Research Centre, Ukkel, Belgium; b Enzootic and (Re)emerging Diseases, Veterinary and Agrochemical Research Centre, Ukkel, Belgium

## Abstract

Pseudorabies virus (PRV) is the causative agent of Aujeszky’s disease in pigs. PRV strains are also used as model organisms for the study of alphaherpesvirus biology or for neuronal pathway studies. We present here the complete genome of the virulent wild-type PRV reference strain NIA3, determined by single-molecule real-time sequencing.

## GENOME ANNOUNCEMENT

Pseudorabies virus (PRV), also called Aujeszky’s disease virus or suid herpesvirus 1, is the causative agent of an economically important disease in the swine industry (1). PRV belongs to the family *Herpesviridae*, subfamily *Alphaherpesvirinae*, genus *Varicellovirus*. It constitutes an excellent model for the study of alphaherpesvirus biology, vaccine development, and neurovirulence (2). A highly virulent field strain NIA3 isolated in Northern Ireland in the early 1970s (3) has frequently been used as a reference PRV strain (4–7). Partial sequences of NIA3 compose the first annotated sequence of a PRV genome, a mosaic of six different PRV strains (8). Here, we obtained the complete NIA3 genome using single-molecule real-time sequencing, a technology that allows robust sequencing and assembling of G+C-rich sequences with repetitive contents.

DNA was purified from virions grown in swine testicle (ST) cells using the Puregene core kit A (Qiagen) according to the manufacturer’s instructions. Genomic DNA was sheared into ~10- to 15-kb fragments for PacBio library preparation and size selected on a BluePippin (Sage Science) using a lower size cutoff of 4 kb. P6-C4 sequencing was performed on 1 single-molecule real-time (SMRT) cell on a PacBio RSII sequencer (Pacific Biosciences) at the Genomics Core UZ Leuven (Belgium).

The SMRT cell generated 46,143 reads (*N*_50_ size 17,528 bp and mean read length 12,777 bp) that were *de novo* assembled into a gapless contig using the HGAP/Quiver-protocol (default parameters, except that minimum seed read length = 17,500; Pacific Biosciences) in SMRT Portal (Pacific Biosciences) version 2.3.0 (9). This contig was polished using consecutive rounds of read mapping with the RS.Resequencing.1 module, resulting in a final assembly with 100% concordance to the reference and an extremely high mean coverage (2,198×) throughout the viral genome. The protein-coding genes were predicted by GeneMarkS (10) and by GATU relative to reference sequence NC_006151.1 (11).

The complete genome of NIA3 is a 142,228 bp long double-stranded linear DNA molecule, with an average G+C content of 73.74%. The long unique and short unique (U_S_) regions are 101,109 and 8,713 bp in size, respectively. The inverted and terminal repeated regions flanking the U_S_ are both 16,203 bp in size. Similar to other PRV genomes, a total of 69 protein-coding genes are identified.

### Nucleotide sequence accession number.

The complete genome of the PRV strain NIA3 was assigned DDBJ/EMBL/GenBank accession no. KU900059.
